# Effect of Cooking Methods on the Antioxidant Capacity of Foods of Animal Origin Submitted to In Vitro Digestion-Fermentation

**DOI:** 10.3390/antiox10030445

**Published:** 2021-03-13

**Authors:** Beatriz Navajas-Porras, Sergio Pérez-Burillo, Álvaro Valverde-Moya, Daniel Hinojosa-Nogueira, Silvia Pastoriza, José Ángel Rufián-Henares

**Affiliations:** 1 Departamento de Nutrición y Bromatología, Instituto de Nutrición y Tecnología de Alimentos, Centro de Investigación Biomédica, Universidad de Granada, 18071 Granada, Spain; beatriznavajas@ugr.es (B.N.-P.); spburillo@ugr.es (S.P.-B.); alvjvm@correo.ugr.es (Á.V.-M.); dhinojosa@ugr.es (D.H.-N.); spdelacueva@ugr.es (S.P.); 2 Instituto de Investigación Biosanitaria ibs.GRANADA, Universidad de Granada, 18071 Granada, Spain

**Keywords:** antioxidant capacity, thermal processing, animal origin food, in vitro digestion, in vitro fermentation, gut microbiota

## Abstract

The human body is exposed to oxidative damage to cells and though it has some endogenous antioxidant systems, we still need to take antioxidants from our diet. The main dietary source of antioxidants is vegetables due to their content of different bioactive molecules. However, there are usually other components of the diet, such as foods of animal origin, that are not often linked to antioxidant capacity. Still, these foods are bound to exert some antioxidant capacity thanks to molecules released during gastrointestinal digestion and gut microbial fermentation. In this work, the antioxidant capacity of 11 foods of animal origin has been studied, submitted to different culinary techniques and to an in vitro digestion and gut microbial fermentation. Results have shown how dairy products potentially provide the highest antioxidant capacity, contributing to 60% of the daily antioxidant capacity intake. On the other hand, most of the antioxidant capacity was released during gut microbial fermentation (90–98% of the total antioxidant capacity). Finally, it was found that the antioxidant capacity of the studied foods was much higher than that reported by other authors. A possible explanation is that digestion–fermentation pretreatment allows for a higher extraction of antioxidant compounds and their transformation by the gut microbiota. Therefore, although foods of animal origin cannot be compared to vegetables in the concentration of antioxidant molecules, the processes of digestion and fermentation can provide some, giving animal origin food some qualities that could have been previously unappreciated.

## 1. Introduction

Global concern about the increased incidence of chronic diseases such as diabetes, obesity, cancer, and cardiovascular disease has led to paying greater attention to lifestyle habits, especially diet [1]. On the other hand, the consumption of animal origin foods has often been linked to the appearance of non-communicable diseases, particularly the consumption of red meat, processed meat, and meat derivatives [2,3]. In contrast, the consumption of plant origin foods, such as fruit and vegetables, has been linked to a protective effect against such conditions [4].

Vegetables’ content in phytochemicals has been pointed out as one of the reasons behind their beneficial effect against such chronic diseases. Many of these compounds have shown great antioxidant activity and thus the potential to play a beneficial role in oxidative stress-related diseases such as cancer, cardiovascular diseases, or type 2 diabetes mellitus [4,5]. At the same time, vegetables’ large and diverse content in biochemicals have made this type of food the object of a large variety of studies [4,5]. In contrast, the literature is very limited in relation to bioactive molecules or antioxidant capacity in animal origin foods such as meat, fish, eggs, or dairy products, probably due to their lack of or low quantities of such molecules, at least in comparison with vegetables. However, we now know that gastrointestinal digestion breaks down food macrostructure and helps to release smaller molecules, some of which could have antioxidant potential [6]. Such is the case of carnosine, a di-peptide with antioxidant activity as well as anti-inflammatory, neuroprotective, and anti-aging properties [7,8]. Therefore, other potentially antioxidant or bioactive molecules are bound to be released during digestion. In addition, other compounds with antioxidant capacity can be found in foods of animal origin, such as taurine [9] and carotenoids from animal feed [10,11].

On the other hand, undigested food passes into the large intestine, where it can be used by the gut microbiota as a fermentation substrate; such undigested food can produce compounds with biological and antioxidant activity [12]. Therefore, although food of animal origin is not characterized by a high content of bioactive molecules, it is still possible that after cooking, digestion, and fermentation, these can be generated. Additionally, cooking methodology will modify, to some degree, depending on the temperature and time applied, the chemical composition of foods. Therefore, gastrointestinal digestion and gut microbial fermentation are likely to be affected and, so too, the molecules released after such processes [13].

Accordingly, the aim of the present paper was to study the antioxidant capacity of animal origin foods, representing the main dietary categories. Different heat treatments were applied, and then they were in vitro digested and fermented. Next, the contribution of the consumption of animal origin foods to the daily intake of antioxidant capacity in Spain was calculated. Finally, the overall daily antioxidant capacity intake in Spain was calculated, also taking into account the antioxidant capacity of plant foods previously studied [14]. 

## 2. Materials and Methods 

### 2.1. Chemicals

#### 2.1.1. In Vitro Digestion and Fermentation

Cysteine, sodium di-hydrogen phosphate, sodium sulphide, resazurin, salivary α-amylase, and pepsin from porcine bile acids (porcine bile extract) were provided by Sigma-Aldrich (Darmstadt, Germany). Pancreatin from porcine pancreas was provided by Alpha Aesar (Lancaster, UK).

#### 2.1.2. Antioxidant Capacity

DPPH (2,2-diphenyl-1-1-picrythydrazyl), hydrochloric acid, iron (III) chloride hexahydrate, methanol, sodium acetate, TPTZ (2,4,6-Tri(2-pyridyl)-s-triazine) and Trolox ((±)-6-Hydroxy-2,5,7,8-tetramethylchromane-2-carboxylic acid) were provided by Sigma- Aldrich (Darmstadt, Germany).

### 2.2. Samples and Cooking Conditions Applied

Eleven animal foods were investigated belonging to the following groups: dairy, egg, fish, and meat (Table S1). Animal foods were bought in three different supermarkets (Carrefour, Dani and El Corte Inglés, Granada, Spain) and stored at room temperature (eggs) or under refrigeration for a maximum of 2 days before cooking.

The foods were submitted to different culinary treatments: boiling, frying, grilling, or roasting (Table S1). Some of them (butter, yogurt, and salmon) were also analyzed in their raw form (since they are usually consumed as raw), making it a total of 36 samples. Boiling was prepared at a rate of 5:1 (water: food) at 100 °C for 20 min. Frying and grilling used Extra virgin olive oil (EVOO) as cooking medium. Frying was prepared at a rate of 5:1 (oil:food) at 180 °C for 8 min. Grilling was prepared at a rate of 0.5:1 (oil:food) at 220–250 °C for 3 min. Roasting was prepared at 180 °C for 10 min. Finally, milk was commercially processed by ultra-high temperature (UHT). Cooking times and food:medium rates were acquired from Olmedilla-Alonso et al. [3] and adapted to our own equipment and laboratory conditions. 

The utensils used for sample preparation were the following: a transportable oven (1500 W), fryer, frying pan and saucepan and forks, knives, spoons, and stainless steel. All these utensils were purchased from Centro Hogar Sánchez (Granada, Spain). Samples were homogenized and stored under nitrogen atmosphere at −80 °C in order to avoid oxidation. All analyses were carried out in duplicate.

### 2.3. In Vitro Digestion and Fermentation

Samples were subjected to an in vitro gastrointestinal and to an in vitro fermentation according to the protocol previously described [15], in triplicate. Food was added to falcon tubes together with simulated salivary fluid (1:1, *w/v*) composed of salts and α-amylase (75 U/mL). The mix was kept at 37 °C for 2 min in oscillation. Right after, 10 mL of simulated gastric fluid was added, simulating the gastric juices content in salts and pepsin (2000 U/mL). The mix was kept at 37 °C for 2 h, at pH 3 in oscillation. Finally, 20 mL of simulated intestinal fluid was added, simulating the intestinal juices content in salts, bile salts, and enzymes (here, we used 67.2 mg/mL pancreatine). The mix was kept at 37 °C for 2 h, at pH 7, in oscillation. Once the intestinal phase was finished, tubes were kept in ice to stop enzymatic reactions and thereafter centrifuged at 3500 rpm for 10 min. The supernatant, which represents the fraction available for absorption in the small intestine, was stored in 1 mL tubes at −80 °C until analysis. The solid pellet, which represents the not digested fraction that goes into the large intestine, was used as in vitro fermentation substrate. 

The in vitro fermentation was carried out using fecal samples from five healthy donors with no previous pathology, who had not taken antibiotics for three months prior to the assay, with a mean (Body Mass Index = 21.3). Individual diets were not assessed since the objective was not to evaluate microbial communities but rather to unravel the potential antioxidant power that average people could extract from animal origin foodstuffs. The fecal samples were pooled together to reduced inter-individual variability. The fermentation was carried out at 37 °C for 20 h. Once the in vitro fermentation was finished, tubes were kept in ice to stop microbial reactions and thereafter centrifuged at 3500 rpm for 10 min. The supernatant, which represents the fraction available for absorption in the large intestine, was stored in 1 mL tubes at −80 °C until analysis. The solid pellet, which represents the fraction not fermented and excreted with feces, was appropriately discarded. 

Therefore, two fractions were obtained after in vitro gastrointestinal digestion and fermentation: digestion supernatant (fraction for absorption in the small intestine), and fermentation supernatant (fraction for absorption in the large intestine). Antioxidant capacity was measured in both fractions, considering as total antioxidant capacity the sum of them. 

### 2.4. Antioxidant Test

Antioxidant capacity of those two fractions was studied. The total antioxidant capacity of the two fractions was taken as the amount of total antioxidant capacity exerted by a given food. [16]. 

*TEAC_DPPH_ assay (Trolox equivalent antioxidant capacity against DPPH radicals).* The method was based on the protocol of Rapisarda et al. [17] and adjusted to a microplate reader (FLUOStar Omega, BMG Labtech, Offenburg, Germany). Briefly, 280 μL of DPPH reagent (prepared with 74 mg DPPH/L methanol) and 20 μL of digestion-fermentation supernatants were added to a 96-well plate. The antioxidant response was monitored in triplicate for one hour at 37 °C. The calibration curve was made up with Trolox at concentrations ranging from 0.01 to 0.4 mg/mL (results expressed as mmol Trolox equivalent/Kg feed).

*TEAC_FRAP_ assay (Trolox equivalent antioxidant capacity referred to reducing capacity)*. The method followed the protocol of Benzie and Strain [18] to measure the ferric reducing capacity in each sample in a microplate reader (FLUOStar Omega, BMG Labtech, Offenburg, Germany). Briefly, 280 μL of FRAP reagent (prepared daily) and 20 μL of digestion-fermentation supernatants were added to a 96-well plate. The antioxidant reaction was followed in triplicate for 30 min at 37 °C. A calibration curve was prepared with Trolox (0.01–0.4 mg/mL), and the results were expressed as mmol Trolox equivalent/Kg feed.

### 2.5. Daily Antioxidant Intake Calculations

The contribution of each food group to daily dietary antioxidant capacity intake was calculated based on the amount of food per serving, the daily intake [18], and the antioxidant capacity previously measured in the samples. The antioxidant capacity of each food was related to the portion size commonly consumed in Spain [19]. Then, the overall daily antioxidant capacity intake was also studied, including both the consumption of foods of animal and plant origin. The data on antioxidant capacity provided by foods of plant origin were obtained from our previous work [20].

### 2.6. Statistical Analysis

The statistical significance of the results was checked by one-way analysis of variance (ANOVA) and subsequently by the Duncan test (*p* < 0.05). As issue for ANOVA, it had been used form of cooking (boiled, fried, grilled, raw, and roasted), sort of food (dairy, egg, fish, and meat) and sort of sample (dairy: butter, cheese, milk and yoghurt; fish: cod fish and salmon; meat: beef, chicken, lamb, and pork). Statistical analysis was performed by using boiled or raw foods and mean of all food groups because the reference groups. Pearson parametric statistic was calculated to indicate the lineal relation between antioxidant capacity at a *p* value < 0.05. To get the significance between the various levels among an equivalent group, the Tukey test was assigned. All the statistical analyses were performed by using Statgraphics Plus software, version 5.1.

## 3. Results

For each sample, the antioxidant capacity was measured in the supernatant fraction obtained after gastrointestinal digestion (antioxidant capacity available for absorption in the small intestine) and after fermentation (antioxidant capacity available for absorption in the large intestine). Two different antioxidant assays were applied. All antioxidant capacity values were corrected, taking into account the antioxidant capacity provided by enzymes, chemicals, and fecal inoculum.

In addition, a linear correlation was obtained by the Spearman method between the two methods. The correlation was significant (*p* < 0.005), with Spearman’s rank correlation coefficient (r_s_) around 0.8.

### 3.1. Samples by Type of Cooking

The types of cooking compared were boiled, fried, grilled, roasted, and UHT. They were compared with each other as well as with respect to the raw food (Table S2).

#### 3.1.1. Gastrointestinal Digestion Supernatant

**Figure 1:**
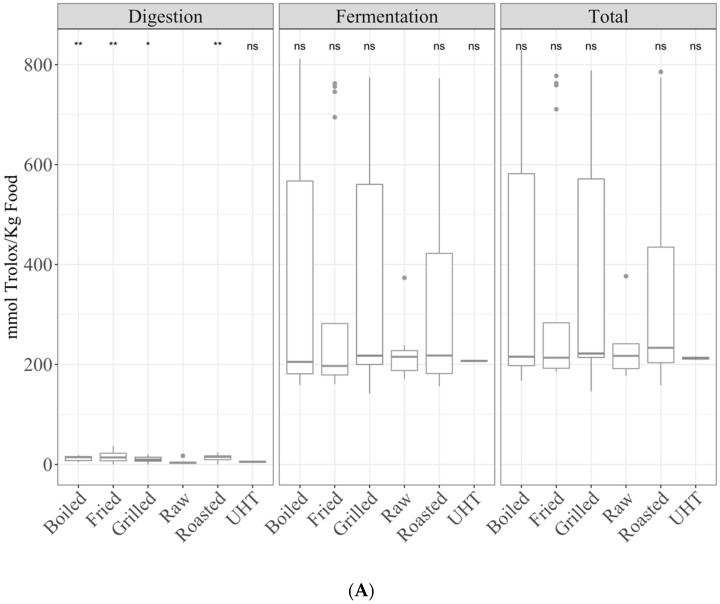

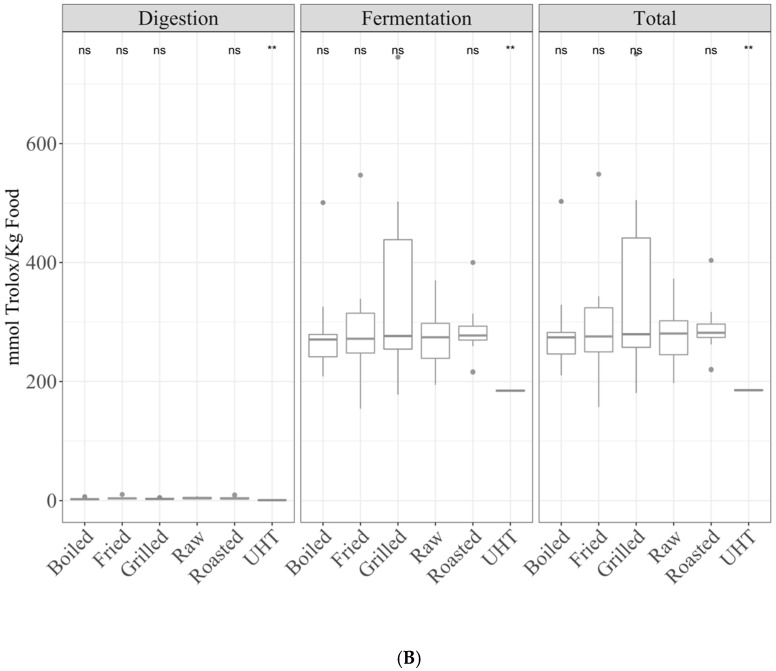
Antioxidant capacity of food of animal origin (butter, cheese, milk, yogurt, egg, cod fish, salmon, beef, chicken, lamb, and pork) obtained after in vitro digestion and fermentation, depending on the cooking technique ((**A**) Trolox capacity against DPPH radicals (TEAC_DPPH_), (**B**) for Trolox equivalent antioxidant capacity referred to reducing capacity (TEAC_FRAP_)). Statistical analysis was performed through ANOVA using raw foods as the reference group. Statistic labels: *: *p* < 0.05, **: *p* < 0.01, ns: not significant.

Regarding TEAC_DPPH_, raw foods showed significantly (*p* < 0.05) lower antioxidant capacity than all types of cooking, except for UHT, which was not significant (Figure 1A). For TEAC_FRAP_, the antioxidant capacity was significantly (*p* < 0.05) lower in UHT foodstuffs than that of raw foods, but no significance was found for the other types of cooking (Figure 1B). In addition, when comparing the means of the different cooking methods, statistically significant differences were found (ANOVA paired comparison; *p* < 0.05; TEAC_DPPH_) for fried foods, being more antioxidant than raw foods.

#### 3.1.2. Fermentation Supernatant and Total Antioxidant Capacity

Regarding TEAC_DPPH_, there were no significant differences (Figure 1A). TEAC_FRAP_ of UHT showed a significantly (*p* < 0.05) lower antioxidant capacity than raw foods (Figure 1B). No other differences with raw foods were found.

In addition, when comparing the means of the different cooking methodologies, the following significant differences were found (ANOVA paired comparison; *p* < 0.05): for TEAC_DPPH_, raw foods were more antioxidant than boiled; for TEAC_FRAP_ UHT were less antioxidant than the rest of cooked foods except roast ones. For both fractions and for the total antioxidant capacity, the significance in ANOVA paired comparison for TEAC_FRAP_, stated that UHT foods were less antioxidant.

### 3.2. Samples by Type of Food

The samples to be compared were divided into four groups: dairy products (composed of butter, cheese, milk and yogurt), eggs, meats (including beef, chicken, lamb, and pork) and fish, which included salmon and cod fish (Table S3).

#### 3.2.1. Gastrointestinal Digestion Supernatant

**Figure 2:**
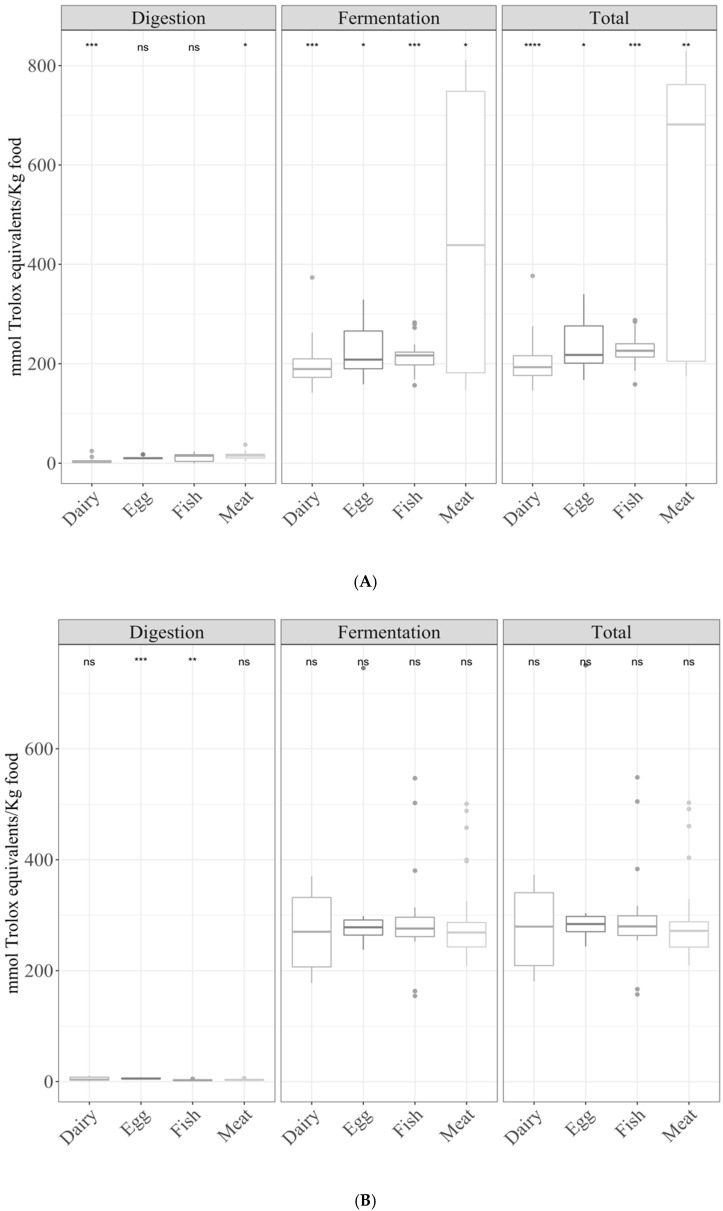
Antioxidant capacity of foods of animal origin (butter, cheese, milk, yogurt, egg, cod fish, salmon, beef, chicken, lamb, and pork) obtained after in vitro digestion and fermentation, depending on the food group ((**A**) TEAC_DPPH_ and (**B**) TEAC_FRAP_). Statistical analysis was performed via ANOVA using the mean antioxidant capacity of all food groups as the reference group. Statistic labels: *: *p* < 0.05, **: *p* < 0.01, ***: *p* < 0.001, ****: *p* < 0.0001, ns: not significant.

Regarding TEAC_DPPH_, meat showed a significantly (*p* < 0.05) higher antioxidant capacity than the rest of the groups. On the other hand, the antioxidant capacity of dairy products was significantly lower than the average antioxidant capacity of the other food groups (Figure 2A). Secondly, for TEAC_FRAP_, the antioxidant capacity of fish was significantly (*p* < 0.05) lower to the other food groups, while that of eggs was the highest (Figure 2B).

#### 3.2.2. Fermentation Supernatant and Total Antioxidant Capacity

In the case of TEAC_DPPH_, the fermentation supernatant and total antioxidant capacities were significantly (ANOVA paired comparison; *p* < 0.05) higher in meat, whereas they were lower in dairy products, egg, and fish compared with the mean antioxidant capacity of all food groups (Figure 2A). For the TEAC_FRAP_ method, there were no significant differences.

**Figure 3:**
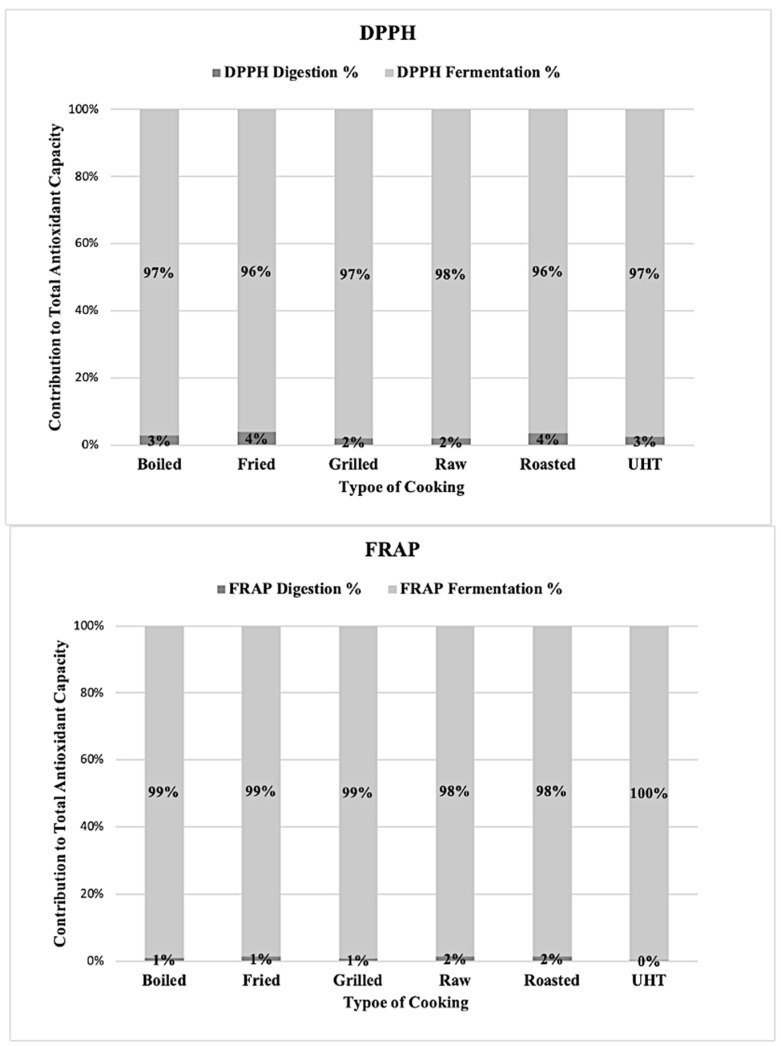
Contribution to the total antioxidant capacity of the fractions obtained after in vitro digestion depending of the cooking technique with the two antioxidant assays.

Figure 3 shows the contribution of each fraction to the total antioxidant capacity. For both methods, the contribution of the digestion fraction was negligible or non-existent, with the fermentation fraction being the most important one.

### 3.3. Specific Group Analysis

The antioxidant capacity within each of the above-mentioned food groups (dairy, fish, and meat) was also analyzed. Each group was studied by cooking method and by type of food. Dairy consisted of butter, cheese, milk, and yoghurt; fish consisted of cod fish and salmon and meat consisted of beef, chicken, lamb, and pork (Table S1).

#### 3.3.1. Dairy

**Figure 4:**
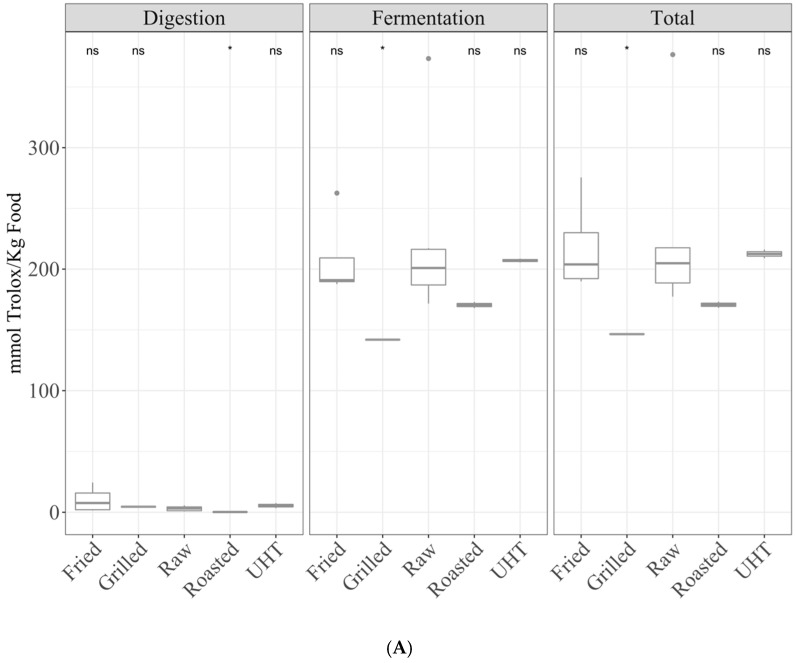

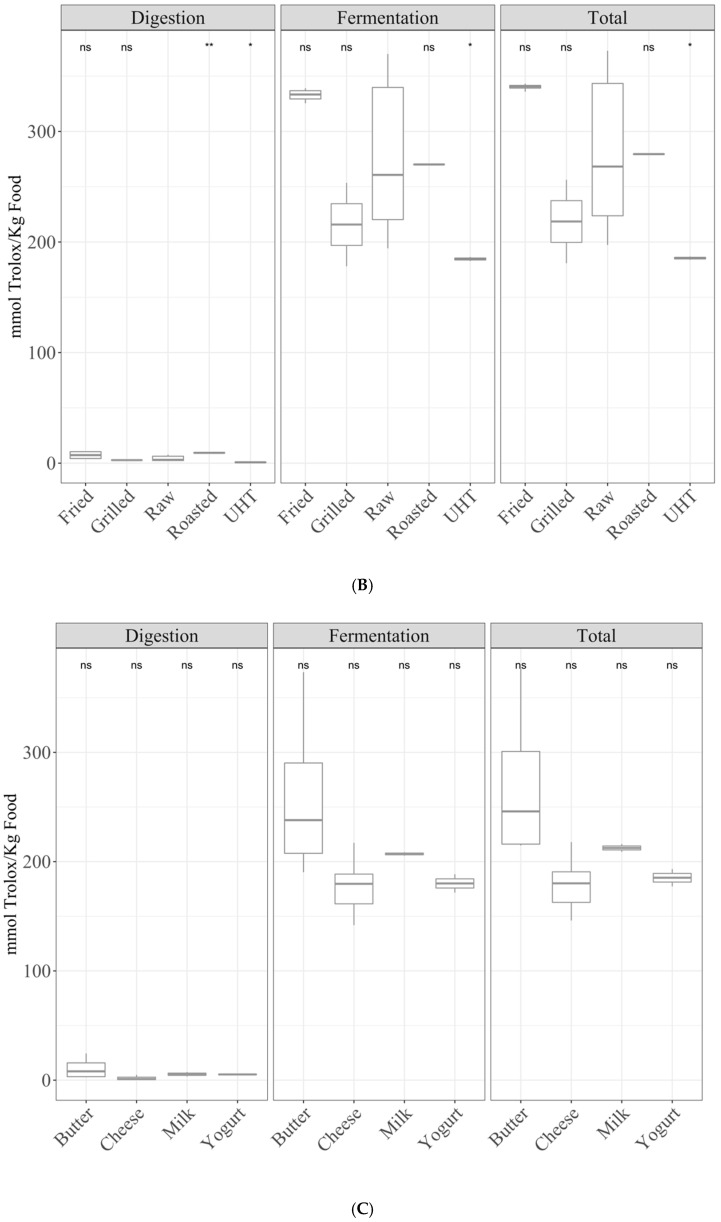

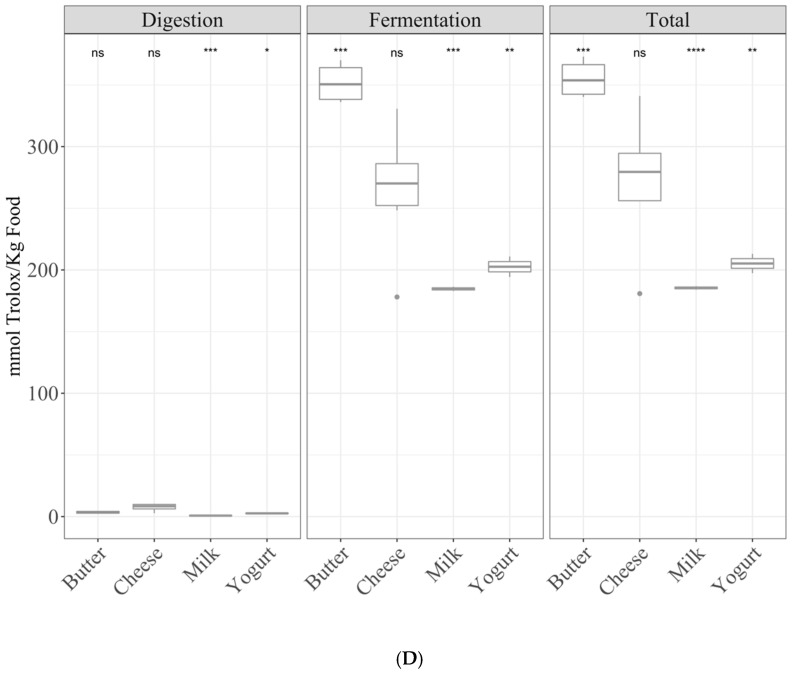
Antioxidant capacity of digested-fermented daily products (butter, cheese, milk and yogurt) depending on the cooking technique ((**A**) TEAC_DPPH_, (**B**) TEAC_FRAP_) and depending on the sample ((**C**) TEAC_DPPH_, (**D**) TEAC_FRAP_). Statistical analysis was performed through ANOVA using raw vegetables to figures A and B or mean of all food groups to figures C and D as the reference group. Statistic labels: *: *p* < 0.05, **: *p* < 0.01, ***: *p* < 0.001, ****: *p* < 0.0001, ns: not significant.

**By cooking** (Table S4). Regarding TEAC_DPPH_ (Figure 4A), raw dairy products showed higher antioxidant capacity than roasted ones in the digestion fraction. However, raw products showed a significantly (*p* < 0.05) higher antioxidant value than grilled products in the fermentation fraction, as well as a higher total antioxidant capacity. Regarding the TEAC_FRAP_ method (Figure 4B), digestion of raw products resulted in a significantly higher antioxidant capacity than UHT, but lower than roasted foods. On the other hand, fermentation of raw products released significantly more antioxidant power than UHT, which resulted as well in a higher total antioxidant capacity.

**By sample** (Table S5). In the case of TEAC_DPPH_ (Figure 4C), comparing the means of the different dairy products (ANOVA paired comparisons, *p* < 0.05), butter antioxidant capacity was higher than that of cheese in the fermented fraction and total antioxidant capacity; for TEAC_FRAP_ (Figure 4D), milk and yogurt were less antioxidant than the other dairy products for the fermented fraction and total antioxidant capacity.

#### 3.3.2. Fish

**Figure 5:**
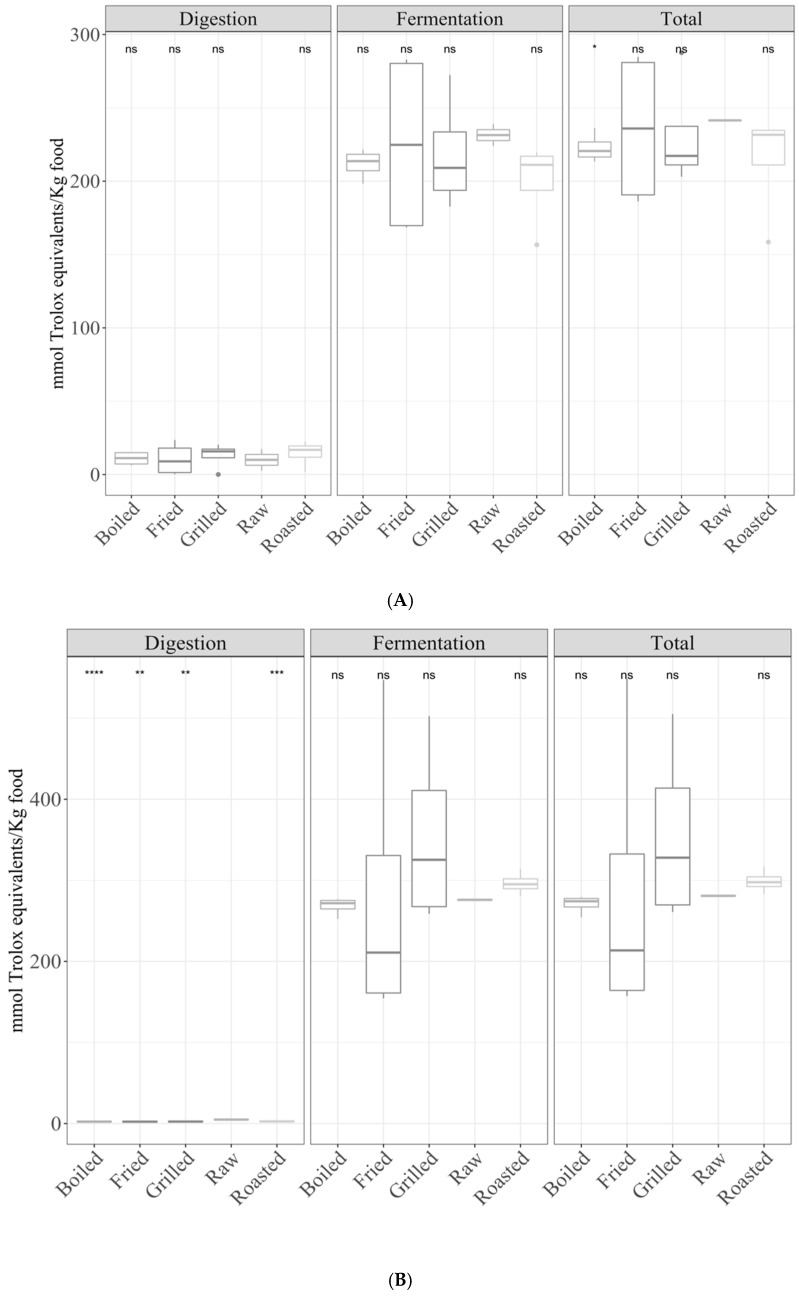

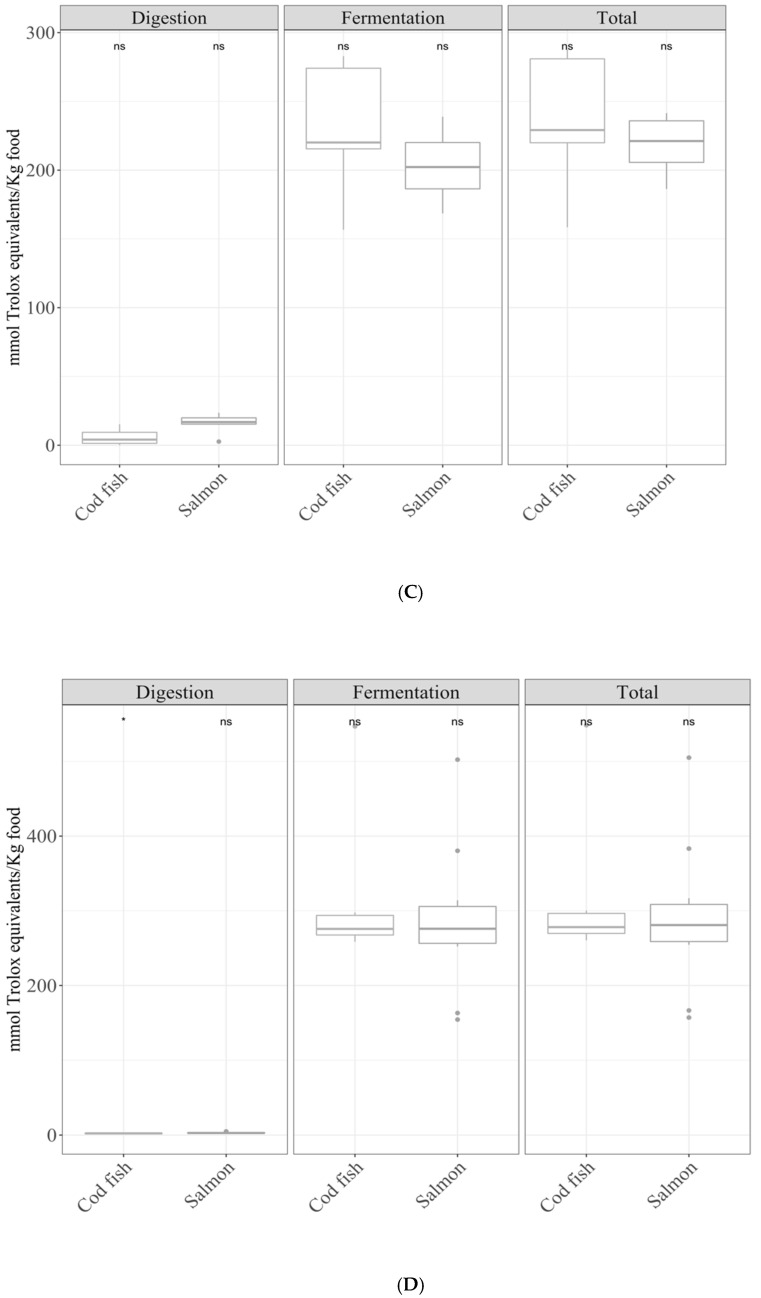
Antioxidant capacity of digested-fermented fish (cod fish and salmon) depending on the cooking technique ((**A**) TEAC_DPPH_, (**B**) TEAC_FRAP_) and depending on the sample ((**C**) TEAC_DPPH_, (**D**) TEAC_FRAP_). Statistical analysis was performed through ANOVA using raw vegetables or mean of all food groups as the reference group. Statistic labels: *: *p* < 0.05, **: *p* < 0.01, ***: *p* < 0.001, ****: *p* < 0.0001, ns: not significant.

**By cooking** (Table S6). No significant differences were found for the TEAC_DPPH_ assay (Figure 5A); for TEAC_FRAP_ (Figure 5B), the digested fraction of raw fish was more antioxidant than cooked ones when comparing the means of the different samples (ANOVA paired comparisons, *p* < 0.05). In the case of the fermented fraction and total antioxidant capacity, there were no significant differences, only for TEAC_DPPH_, where boiled fish was less antioxidant than raw.

On the other hand, **by sample** (Table S7), in the case of TEAC_DPPH_ (Figure 5C), when comparing the means of the different samples (ANOVA paired comparisons, *p* < 0.05), salmon (blue fish) was more antioxidant than cod fish (white fish) after digestion; for the TEAC_FRAP_ method (Figure 5D), salmon (blue fish) was the most antioxidant foodstuff when comparing means of different samples (ANOVA paired comparisons, *p* < 0.05).

#### 3.3.3. Meat

**Figure 6:**
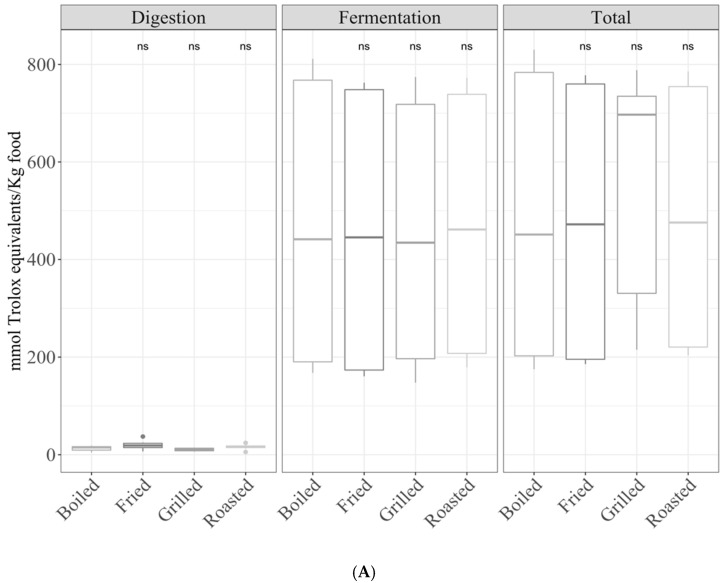

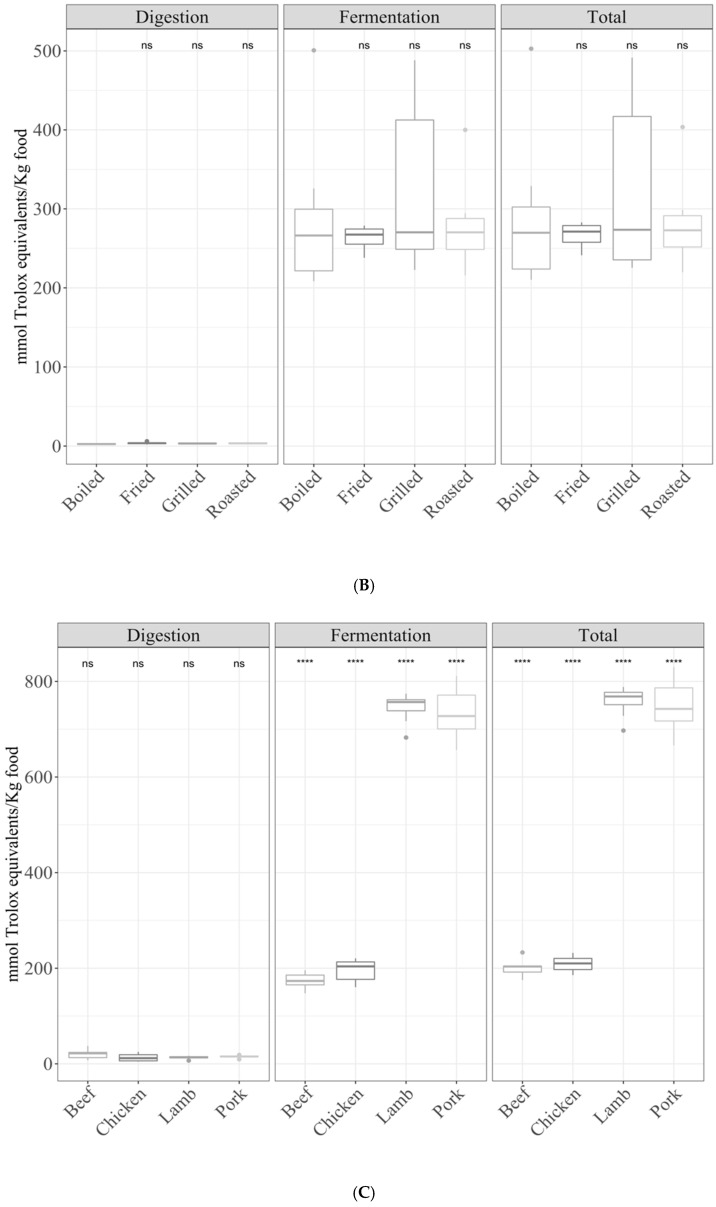

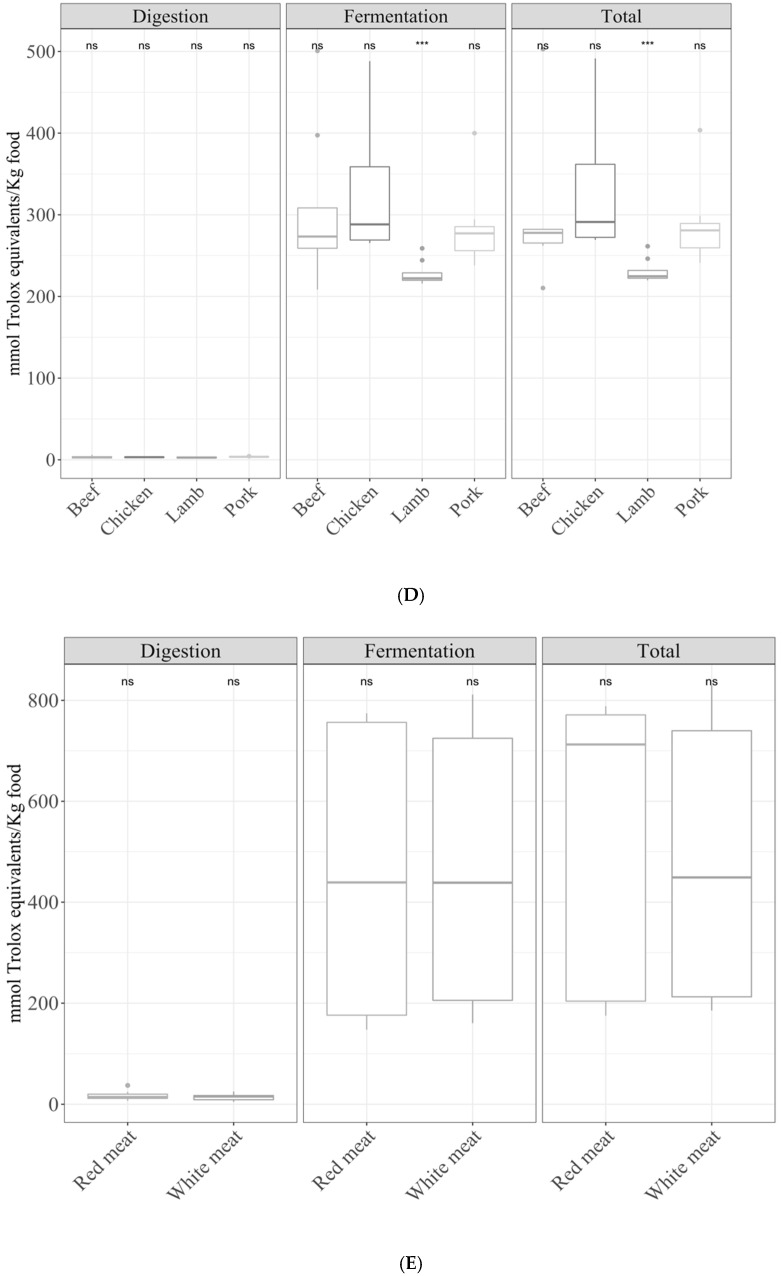

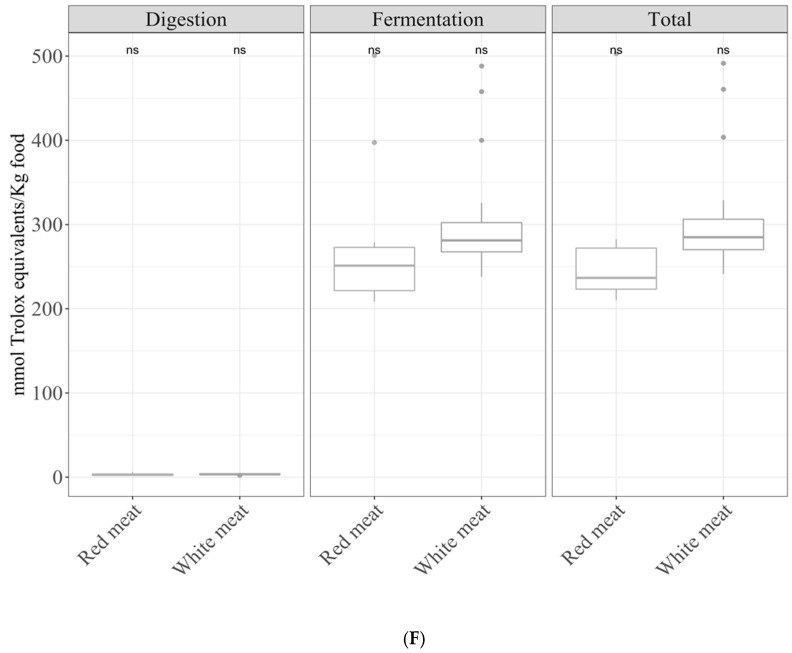
Antioxidant capacity of digested-fermented meat (beef, chicken, lamb, and pork) depending on the cooking technique ((**A**) TEAC_DPPH_, (**B**) TEAC_FRAP_), depending on the sample ((**C**) TEAC_DPPH_, (**D**) TEAC_FRAP_) and depending of the type of meat, red or white ((**E**) TEAC_DPPH_, (**F**) TEAC_FRAP_). Statistical analysis was performed through ANOVA using raw vegetables or mean of all food groups as the reference group. Statistic labels: ***: *p* < 0.001, ****: *p* < 0.0001, ns: not significant.

No significant differences were found in meat **by cooking** (Table S8), either for TEAC_DPPH_ (Figure 6A) or for TEAC_FRAP_ (Figure 6B). On the other hand, **by sample** (Table S9), for TEAC_DPPH_ (Figure 6C) lamb and pork were significantly more antioxidant than beef and chicken after fermentation, as well as the total antioxidant capacity. In the case of TEAC_FRAP_ (Figure 6D) the antioxidant capacity of chicken was higher than that of lamb, both total antioxidant capacity and after in vitro fermentation. Differences between red and white meat were analyzed (Table S10) and not many significant differences were observed (Figure 6E,F).

The antioxidant capacities of meats and fish were also compared. In this sense, fish showed significantly lower antioxidant capacity (TEAC_DPPH_) than meat in the fermentation fraction and total antioxidant capacity.

### 3.4. Daily Antioxidant Intake

We first calculated the contribution of animal food consumption to the daily antioxidant capacity intake, taking into account just the consumption of food of animal origin (Table 1 and Table 2), so that their sum reaches 100%. Dairy products showed the highest contribution to the daily antioxidant capacity intake in the Spanish diet, ranging between 56% (DPPH assay) and 66% (FRAP assay) of the antioxidant capacity provided by foods of animal origin. Meats also stood out with a contribution of 35% (DPPH assay) and 23% (FRAP assay). When we considered the antioxidant capacity computed by portion size, fish contributed with 25% (DPPH assay) and 62% (FRAP assay), whereas meat contributed with 43% (DPPH assay) and 45% (FRAP assay) of the antioxidant capacity (Table 1).

Regarding to the cooking method applied (Table 2), roasted dairy products contributed 18% to the daily antioxidant capacity coming from foods of animal origin (DPPH assay), and raw dairy products 19% (FRAP assay). Taking into account the consumption portion, roasted meat contributed up to 32% of the daily antioxidant capacity (DPPH assay) derived from an animal source, while grilled-roasted fish contributed 29% (FRAP assay).

The contribution of food consumption to the daily antioxidant intake was also studied, taking into account the total diet, including also vegetable foods (Table 3) using for calculations also our results recently published regarding this type of food [14]. Taking into consideration the main food groups of the Spanish diet, it is noteworthy to mention that dairy products (35% in DPPH assay and 28% in FRAP assay) and meat (12% in DPPH assay and 18% in FRAP assay) were the most antioxidant foods when the daily intake was computed. If the serving size were used, the contribution to the daily antioxidant capacity was slightly modified for meat (24% in DPPH assay and 40% in FRAP assay) and fish (32% in DPPH assay and 23% in FRAP assay). Thus, in the case of the DPPH method, the top five food groups contributing to the daily antioxidant intake per serving were fish > egg > meat tubers > fruits. In the case of the FRAP method: meat > fish > egg > fruits > tubers.

## 4. Discussion

In most cases, heat treatment positively affects the antioxidant capacity of food [21,22,23]. In this study, foods subjected to different cooking techniques were compared with their raw form. It was found that cooking generally increased the antioxidant capacity of foods, especially fried foods. Similar results have been found in other studies [24,25,26] that claim that olive oil used for frying provides a high antioxidant capacity to the preparation. However, some cooking techniques, such as boiling, could result in a loss of hydrosoluble compounds in the cooking water, such as B vitamins, and therefore antioxidant capacity could be reduced [21].

The highest antioxidant capacity was obtained after in vitro fermentation of foods (more than 90% of the total antioxidant capacity). This is an important result of our study, since in vitro fermentation potentially release-transform bioactive compounds with high antioxidant capacity. Therefore, the gut microbiota seems to play an important role in the release of these compounds from the indigestible matrix of animal-derived foods [24,25], as in the case of plant-derived foods [14]. Heat treatment catalyzes different chemical reactions such the Maillard reaction [27,28,29]. In this sense, cooking techniques with a high heat-load (i.e., frying, grilling, and roasting) can produce a large amount of melanoproteins [30,31], which are end-products of the Maillard reaction with a high antioxidant capacity [32]. Such melanoidins are hardly digested and reach the colon, where they are metabolized by the gut microbiota [33].

The antioxidant capacity of digested meats (beef, chicken, lamb, and pork) ranged from 13.2 to 20.5 mmol Trolox equivalents/Kg meat (Table S10), which is in line with values reported by other authors [26]. However, the study reported by Carrillo et al. [26] doesn’t include the antioxidant capacity obtained after in vitro fermentation, which is up to 95% higher, reinforcing the idea that the fermentation step is needed to check the overall antioxidant potential of a given food. Lamb and pork meats were the most antioxidant meats with the DPPH method, while lamb was the lowest one with the FRAP assay (Table S10). This could be related to the poor ability of lamb antioxidants to reduce ferric ion to its ferrous form instead of quenching radical species [26]. In addition, although the antioxidant capacity of digested meat and fish was similar (Table S3) the final antioxidant capacity of meat was higher, since more antioxidant compounds could be released after fermentation. These differences could come from the feed that these animals have. The feeding of meat-producing animals is more controlled than that of fish, and they may have been fed feeds rich in compounds with antioxidant activity [10].

In the group of dairy products, butter stood out as the food with the greatest antioxidant capacity. This could be explained, taking into account that some antioxidant compounds in dairy products (such as α-tocopherol, β-carotene, vitamins A and D_3_, and phospholipids) are found in milk fat, the main component of butter [11].

Among all the foods chosen for this study, meat stood out for its antioxidant capacity, while dairy products and fish had the lowest values, which doesn’t mean that their contribution to the antioxidant capacity intake with the diet is also lower. The antioxidant capacity provided by each food was studied, taking into account daily consumption in a regular diet [19], as well as portion sizes [20] (Table 1). In Table 2, the culinary treatments applied were also taken into account. Dairy products, which are highly consumed by the Spanish population [19], stood out for their daily intake, as well as roasted meat and grilled fish.

Till now, the efforts on calculating the contribution of the regular diet to the daily antioxidant intake have been centered in plant foods [16,34], since they provide many bioactive antioxidant compounds such as phenolic compounds, vitamins, etc. Thus, our results cannot be compared with other papers on the matter, since there is no scientific literature about the contribution of animal foods to the daily antioxidant capacity. However, foods of animal origin are also a good source of antioxidant compounds like dipeptides (carnosine and anserine), uric acid, polyamines, ascorbic acid, α-tocopherol, B group vitamins, carotenoids, ubiquinone, among others [26]. This is why we calculated the overall contribution of the Spanish diet to the daily antioxidant capacity (Table 3), taking into account the intake of animal origin foods (data reported in the paper) and plant foods [14]. The first interesting result is that the Spanish diet provides an average of 175.1 (DPPH) and 164.3 (FRAP) mmol Trolox/day, which is much higher than that previously reported [34] for vegetable products only (6.1 mmol Trolox/day). This could be explained by taking into account that the initials calculations performed by Saura-Calixto and Goñi [33] were computed with the usual extraction method of antioxidant species, avoiding the large effects of digestion and fermentation. In addition, it is noteworthy to mention that the contribution of animal foods was notable (49.7% and 53.1% of the total antioxidant capacity intake for DPPH and FRAP methods), reaching 87.1 and 87.3 mmol Trolox/day for DPPH and FRAP assays, respectively. The food groups with a higher contribution to the daily antioxidant capacity intake of the Spanish diet were as follows: dairy > cereals > meat > fruits > vegetables > tubers > egg (DPPH) and dairy > meat > cereals > fruits > vegetables > tubers > egg (FRAP). However, if an increase in antioxidant capacity intake should be recommended, them the food groups suggested (due to the high antioxidant capacity provided by a portion) will be: fish > egg > meat > tubers > dairy > vegetables (DPPH) and meat > fish > egg > fruits > tubers > dairy.

## 5. Conclusions

In conclusion, this study reinforces the concept that foods of animal origin could be considered as a good source of antioxidant compounds for humans. This research has demonstrated that though animal origin food may not be rich in bioactive antioxidant components (like plant foods) gastrointestinal digestion and, more importantly, gut microbiota fermentation, can improve the antioxidant properties of such foods. Most of the antioxidant power of these foodstuffs was released subsequent to in vitro gut microbiota fermentation (around 90%). The food groups with the highest antioxidant capacity were meat and fish, which were increased even more after frying and boiling. The foods that contributed the most antioxidant capacity to the diet in terms of daily consumption were dairy products, while in terms of portion size, the foods with the highest antioxidant capacity were meat and fish. Therefore, the daily antioxidant capacity intake in the Spanish diet has been revisited, finding that foods of animal origin contribute to around 50% of the daily antioxidant capacity intake. So, further studies on antioxidant capacity involving foods of animal origin after in vitro digestion and fermentation should be carried out in the future in order to estimate their contribution to the daily intake of antioxidant capacity.

## Figures and Tables

**Table 1:** Contribution of food of animal origin consumption to the daily antioxidant capacity (AOX) intake in the Spanish diet.

**Food Type**	**Analytical Assay**	**AOX/Daily Intake ^1^ (μmol Trolox/day)**	**AOX/Serving Intake ^2^ (μmol Trolox/serving)**	**Mean Contribution to Daily Antioxidant Intake (%)**	**Mean Contribution to Daily Antioxidant Per Serving Intake (%)**
Dairy	*DPPH*	49,170	23,198	56.3	14.1
Egg	*DPPH*	5491	28,871	6.29	17.6
Meat	*DPPH*	31,308	70,944	35.9	43.2
Fish	*DPPH*	1344	41,173	1.54	25.1
**Food Type**	**Analytical Assay**	**AOX/Daily Intake ^1^ (μmol Trolox/day)**	**AOX/Serving Intake ^2^ (μmol Trolox/serving intake)**	**Mean Contribution to Daily Antioxidant Intake (%)**	**Mean Contribution to Daily Antioxidant Per Serving Intake (%)**
Dairy	*FRAP*	57,643	29,660	66.2	34.0
Egg	*FRAP*	7659	40,271	8.79	46.2
Meat	*FRAP*	20,042	39,518	23.0	45.4
Fish	*FRAP*	1765	54,028	2.03	62.0

^1^ Considering consumption for a whole year; ^2^ Considering the intake of 1 serving.

**Table 2:** Contribution of food of animal origin, with different culinary treatments, consumption to the daily antioxidant capacity (AOX) intake in the Spanish diet.

Food Type	Thermal Processing	Analytical Assay	AOX/Daily Intake ^1^ (μmol Trolox/day)	AOX/Serving Intake ^2^ (μmol Trolox/serving)	Mean Contribution to Daily Antioxidant Intake (%)	Mean Contribution to Daily Antioxidant Per Serving Intake (%)
*Dairy*	Fried	DPPH	4319	8539	1.69	3.35
*Dairy*	Raw	DPPH	4670	15,299	1.83	6.00
*Dairy*	Roasted	DPPH	44,700	23,660	17.5	9.28
*Dairy*	Brewed	DPPH	5973	19,564	2.34	7.68
*Egg*	Boiled	DPPH	35,026	46,355	13.7	18.2
*Egg*	Fried	DPPH	5962	31,351	2.34	12.3
*Egg*	Grilled	DPPH	6068	31,908	2.38	12.5
*Egg*	Roasted	DPPH	12,030	63,257	4.72	24.8
*Meat*	Boiled	DPPH	6574	34,568	2.58	13.6
*Meat*	Fried	DPPH	32,686	72,016	12.8	28.3
*Meat*	Grilled	DPPH	30,649	70,579	12.0	27.7
*Meat*	Roasted	DPPH	28,329	82,381	11.1	32.3
*Fish*	Boiled	DPPH	31,625	71,840	12.4	28.2
*Fish*	Fried	DPPH	1320	40,085	0.52	15.7
*Fish*	Grilled	DPPH	1320	41,083	0.52	16.1
*Fish*	Raw	DPPH	1460	44,605	0.57	17.5
*Fish*	Roasted	DPPH	969	50,549	0.38	19.8
*Dairy*	Fried	FRAP	7552	14,410	3.42	6.53
*Dairy*	Raw	FRAP	41,077	23,419	18.6	10.6
*Dairy*	Roasted	FRAP	5973	19,564	2.71	8.87
*Dairy*	UHT	FRAP	35,026	46,355	15.9	21.0
*Egg*	Boiled	FRAP	5962	31,351	2.70	14.2
*Egg*	Fried	FRAP	6068	31,908	2.75	14.5
*Egg*	Grilled	FRAP	12,030	63,257	5.45	28.7
*Egg*	Roasted	FRAP	6574	34,568	2.98	15.7
*Meat*	Boiled	FRAP	21,833	41,983	9.90	19.0
*Meat*	Fried	FRAP	19,589	38,637	8.88	17.5
*Meat*	Grilled	FRAP	24,088	45,616	10.9	20.7
*Meat*	Roasted	FRAP	22,053	40,586	10.0	18.4
*Fish*	Boiled	FRAP	1593	48,692	0.72	22.1
*Fish*	Fried	FRAP	1593	48,939	0.72	22.2
*Fish*	Grilled	FRAP	2191	63,983	0.99	29.0
*Fish*	Raw	FRAP	969	50,549	0.44	22.9
*Fish*	Roasted	FRAP	1770	53,802	0.80	24.4

^1^ Considering consumption for a whole year; ^2^ Considering the intake of 1 serving.

**Table 3:** Antioxidant capacity distributed as a % of each food group in relation to the total diet.

Type of Food	Mean Contribution to Daily Antioxidant Capacity Intake (%) DPPH Assay	Mean Contribution to Daily Antioxidant Capacity Per Serving Intake (%) DPPH Assay	Mean Contribution to Daily Antioxidant Capacity Intake (%) FRAP Assay	Mean Contribution to Daily Antioxidant Capacity Per Serving Intake (%) FRAP Assay
*Dairy*	35.1	18.1	28.1	13.2
*Egg*	4.70	24.5	3.10	16.5
*Meat*	12.2	24.1	17.9	40.5
*Fish*	1.10	32.9	0.80	23.5
*Alcoholic drinks* ^1^	0.70	2.20	4.40	10.1
*Cereals* ^1^	13.6	3.90	12.7	3.40
*Cocoa* ^1^	0.60	4.20	0.60	4.60
*Coffee* ^1^	0.20	0.90	0.60	2.80
*Fruits* ^1^	11.6	13.5	12.1	15.1
*Legumes* ^1^	0.80	10.1	0.70	9.20
*Nuts* ^1^	0.80	3.50	0.70	2.70
*Oils* ^1^	0.30	0.20	1.10	0.60
*Tubers* ^1^	9.00	19.0	6.50	14.3
*Vegetables* ^1^	9.30	9.70	10.7	9.80

^1^ Considering the data of reference [14].

## Data Availability

The data presented in this study are available as supplementary material.

## Supplementary Materials

The following are available online at https://www.mdpi.com/2076-3921/10/3/445/s1, Supplemental Table S1. Food of animal origin and cooking conditions. Supplemental Table S2. Antioxidant capacity of in vitro digested-fermented foods of animal origin depending on the cooking method. Supplemental Table S3. Antioxidant capacity of in vitro digested-fermented foods of animal origin depending on the group. Supplemental Table S4. Antioxidant capacity of in vitro digested-fermented dairy foods depending on the cooking method. Supplemental Table S5. Antioxidant capacity of in vitro digested-fermented dairy foods depending on the dairy type. Supplemental Table S6. Antioxidant capacity of in vitro digested-fermented fish depending on the cooking method. Supplemental Table S7. Antioxidant capacity of in vitro digested-fermented fish depending on the fish type. Supplemental Table S8. Antioxidant capacity of in vitro digested-fermented meat depending on the cooking method. Supplemental Table S9. Antioxidant capacity of in vitro digested-fermented meat depending on the meat type. Supplemental Table S10. Antioxidant capacity of in vitro digested-fermented red and white meat.
