# Supplementary material for: Effect of Cooking Methods on the Antioxidant Capacity of Foods of Animal Origin Submitted to In Vitro Digestion-Fermentation
Source: Antioxidants (Basel). 2021 Mar 13;10(3):445. doi: 10.3390/antiox10030445 (PMC7999583; doi:10.3390/antiox10030445)
Supplement: Supplementary file 1 [file antioxidants-10-00445-s001.pdf]

Effect of cooking methods on the antioxidant capacity of foods of animal origin submitted to in vitro digestion-fermentation

SUPPLEMENTAL INFORMATION

Supporting information description

Supplemental Table 1. Food of animal origin and cooking conditions.

Supplemental Table 2. Antioxidant capacity of *in vitro* digested-fermented foods of animal origin depending on the cooking method.

Supplemental Table 3. Antioxidant capacity of *in vitro* digested-fermented foods of animal origin depending on the group.

Supplemental Table 4. Antioxidant capacity of *in vitro* digested-fermented dairy foods depending on the cooking method.

Supplemental Table 5. Antioxidant capacity of *in vitro* digested-fermented dairy foods depending on the dairy type.

Supplemental Table 6. Antioxidant capacity of *in vitro* digested-fermented fish depending on the cooking method.

Supplemental Table 7. Antioxidant capacity of *in vitro* digested-fermented fish depending on the fish type.

Supplemental Table 8. Antioxidant capacity of *in vitro* digested-fermented meat depending on the cooking method.

Supplemental Table 9. Antioxidant capacity of *in vitro* digested-fermented meat depending on the meat type.

Supplemental Table 10. Antioxidant capacity of *in vitro* digested-fermented red and white meat.

Supplemental Table 1. Food of animal origin and cooking conditions.

Group	Sample name	Cooking method
Dairy	Butter	Fried
Dairy	Butter	Raw
Dairy	Cheese (Gouda)	Fried
Dairy	Cheese (Gouda)	Grilled
Dairy	Cheese (Gouda)	Raw
Dairy	Cheese (Gouda)	Roasted
Dairy	Milk	UHT
Dairy	Yogurt	Raw
Egg	Egg	Boiled
Egg	Egg	Fried
Egg	Egg	Grilled
Egg	Egg	Roasted
Fish	Cod fish	Boiled
Fish	Cod fish	Fried
Fish	Cod fish)	Grilled
Fish	Cod fish	Roasted
Fish	Salmon	Boiled
Fish	Salmon	Fried
Fish	Salmon	Grilled
Fish	Salmon	Raw
Fish	Salmon	Roasted
Meat	Beef	Boiled
Meat	Beef	Fried
Meat	Beef	Grilled
Meat	Beef	Roasted
Meat	Chicken	Boiled
Meat	Chicken	Fried
Meat	Chicken	Grilled
Meat	Chicken	Roasted
Meat	Lamb	Boiled
Meat	Lamb	Fried
Meat	Lamb	Grilled
Meat	Lamb	Roasted
Meat	Pork	Boiled
Meat	Pork	Fried
Meat	Pork	Grilled
Meat	Pork	Roasted

Supplemental Table 2. Antioxidant capacity of *in vitro* digested-fermented foods of animal origin depending on the cooking method.

**Cooking technique**	**$TEAC_{DPPH}$ (µmol Trolox/Kg food)**		
	**Digested fraction**	**Fermented fraction**	**Total antioxidant capacity**
Boiled	$12.5^{a,b} \pm 4.81$	$354^{a} \pm 270$	$368^{a} \pm 272$
UHT	$5.45^{a,b} \pm 2.92$	$207^{a} \pm 2.21$	$213^{a} \pm 5.13$
Fried	$14.5^{b} \pm 10.1$	$322^{a} \pm 233$	$336^{a} \pm 232$
Grilled	$10.2^{a,b} \pm 5.49$	$326^{a} \pm 237$	$361^{a} \pm 59.8$
Raw	$4.76^{a} \pm 5.41$	$227^{a} \pm 63.3$	$231^{a} \pm 63.3$
Roasted	$13.1^{a,b} \pm 7.64$	$347^{a} \pm 240$	$360^{a} \pm 242$
	**$TEAC_{FRAP}$ (µmol Trolox/Kg food)**		
	**Digested fraction**	**Fermented fraction**	**Total antioxidant capacity**
Boiled	$3.07^{a} \pm 1.41$	$277^{a} \pm 71.4$	$280^{a} \pm 71.3$
UHT	$0.77^{a} \pm 0.44$	$185^{a} \pm 2.45$	$185^{a} \pm 2.02$
Fried	$4.29^{a} \pm 2.50$	$282^{a} \pm 83.3$	$287^{a} \pm 83.5$
Grilled	$3.23^{a} \pm 0.95$	$342^{a} \pm 147$	$347^{a} \pm 155$
Raw	$4.41^{a} \pm 2.19$	$276^{a} \pm 63.2$	$281^{a} \pm 62.8$
Roasted	$4.36^{a} \pm 2.37$	$281^{a} \pm 41.1$	$286^{a} \pm 40.9$

Different letters in the same column indicates statistically significant differences ($p < 0.05$)

Supplemental Table 3. Antioxidant capacity of *in vitro* digested-fermented foods of animal origin depending on the group.

**Food**	**TEAC$_{DPPH}$ (µmol Trolox/Kg food)**		
	**Digested fraction**	**Fermented fraction**	**Total antioxidant capacity**
Dairy	4.98^{a} ± 6.12	201^{a} ± 54.5	206^{a} ± 55.2
Egg	10.8^{a,b} ± 2.91	230^{a} ± 66.0	241^{a} ± 65.6
Fish	12.0^{b} ± 8.00	216^{a} ± 36.1	228^{a} ± 33.5
Meat	15.2^{b} ± 6.90	463^{b} ± 284	499^{b} ± 280
Mean	*8.60 ± 5.98*	*278 ± 110*	*294 ± 109*
	**TEAC$_{FRAP}$ (µmol Trolox/Kg food)**		
	**Digested fraction**	**Fermented fraction**	**Total antioxidant capacity**
Dairy	5.03^{a} ± 3.47	271^{a} ± 67.0	276^{a} ± 68.3
Egg	5.68^{a} ± 0.89	330^{a} ± 169	336^{a} ± 169
Fish	2.73^{b} ± 0.89	297^{a} ± 96.6	300^{a} ± 96.4
Meat	3.23^{b} ± 0.87	288^{a} ± 77.3	288^{a} ± 77.1
Mean	*4.18 ± 1.53*	*297 ± 102*	*300 ± 103*

Different letters in the same column indicates statistically significant differences ($p < 0.05$)

Supplemental Table 4. Antioxidant capacity of *in vitro* digested-fermented dairy foods depending on the cooking method.

Cooking technique	DPPH (mmol Trolox equivalents/Kg food)		
	**Digested fraction**	**Fermented fraction**	**Total antioxidant capacity**
UHT	$5.45^{a} \pm 2.92$	$207^{a} \pm 2.21$	$213^{a} \pm 5.13$
Fried	$10.3^{a} \pm 10.8$	$208^{a} \pm 36.4$	$218^{a} \pm 39.7$
Grilled	$4.54^{a} \pm 0.52$	$142^{a} \pm 0.09$	$146^{a} \pm 0.44$
Raw	$3.01^{a} \pm 2.08$	$225^{a} \pm 74.7$	$228^{a} \pm 74.5$
Roasted	$0.22^{a} \pm 0.05$	$170^{a} \pm 3.61$	$171^{a} \pm 3.66$
	**FRAP (mmol Trolox equivalents/Kg food)**		
	**Digested fraction**	**Fermented fraction**	**Total antioxidant capacity**
UHT	$0.77^{a} \pm 0.44$	$185^{a} \pm 2.45$	$186^{a} \pm 2.01$
Fried	$7.30^{a} \pm 3.59$	$333^{a} \pm 6.00$	$340^{b} \pm 3.07$
Grilled	$2.75^{a} \pm 0.00$	$216^{a} \pm 53.4$	$219^{a,b} \pm 53.4$
Raw	$4.25^{a} \pm 2.58$	$276^{a} \pm 74.7$	$281^{a,b} \pm 74.3$
Roasted	$9.36^{a} \pm 0.46$	$270^{a} \pm 0.05$	$280^{a,b} \pm 0.51$

Different letters in the same column indicates statistically significant differences ($p < 0.05$)

Supplemental Table 5. Antioxidant capacity of *in vitro* digested-fermented dairy foods depending on the dairy type.

Sample	DPPH (mmol Trolox equivalents/Kg food)		
	**Digested fraction**	**Fermented fraction**	**Total antioxidant capacity**
Butter	$10.9^{a} \pm 10.1$	$260^{b} \pm 81.4$	$271^{b} \pm 76.0$
Gouda	$1.82^{a} \pm 1.83$	$176^{a} \pm 25.6$	$178^{a} \pm 24.4$
Milk	$5.45^{a} \pm 2.92$	$207^{a,b} \pm 2.21$	$213^{a,b} \pm 5.13$
Yogurt	$5.23^{a} \pm 0.65$	$180^{a,b} \pm 11.9$	$185^{a,b} \pm 11.2$
Mean	*5.85 ± 3.88*	*206 ± 30.3*	*212 ± 29.2*
	**FRAP (mmol Trolox equivalents/Kg food)**		
	**Digested fraction**	**Fermented fraction**	**Total antioxidant capacity**
Butter	$3.39^{a,b} \pm 0.97$	$352^{b} \pm 16.8$	$355^{b} \pm 15.9$
Gouda	$7.51^{b} \pm 3.14$	$269^{a} \pm 47.7$	$276^{a} \pm 50.3$
Milk	$0.77^{a} \pm 0.44$	$185^{a} \pm 2.45$	$186^{a} \pm 2.01$
Yogurt	$2.62^{a,b} \pm 0.65$	$203^{a} \pm 11.8$	$205^{a} \pm 11.1$
Mean	*3.57 ± 1.3*	*252 ± 19.7*	*256 ± 19.8*

Different letters in the same column indicates statistically significant differences ($p < 0.05$)

Supplemental Table 6. Antioxidant capacity of *in vitro* digested-fermented fish depending on the cooking method.

Cooking technique	DPPH (mmol Trolox equivalents/Kg food)		
	**Digested fraction**	**Fermented fraction**	**Total antioxidant capacity**
Boiled	$10.9^{a} \pm 4.69$	$212^{a} \pm 10.1$	$223^{a} \pm 9.96$
Fried	$10.4^{a} \pm 11.3$	$225^{a} \pm 64.6$	$236^{a} \pm 53.8$
Grilled	$13.0^{a} \pm 8.90$	$218^{a} \pm 39.3$	$231^{a} \pm 38.2$
Raw	$10.0^{a} \pm 10.5$	$231^{a} \pm 10.5$	$241^{a} \pm 0.05$
Roasted	$14.5^{a} \pm 8.94$	$200^{a} \pm 29.2$	$214^{a} \pm 37.2$
	**FRAP (mmol Trolox equivalents/Kg food)**		
	**Digested fraction**	**Fermented fraction**	**Total antioxidant capacity**
Boiled	$2.38^{a} \pm 0.09$	$268^{a} \pm 11.1$	$271^{a} \pm 11.2$
Fried	$2.39^{a} \pm 0.84$	$281^{a} \pm 184$	$283^{a} \pm 184$
Grilled	$2.45^{a} \pm 0.44$	$353^{a} \pm 114$	$355^{a} \pm 114$
Raw	$4.89^{b} \pm 0.02$	$276^{a} \pm 0.58$	$281^{a} \pm 0.59$
Roasted	$2.63^{a} \pm 0.18$	$296^{a} \pm 13.8$	$299^{a} \pm 13.9$

Different letters in the same column indicates statistically significant differences ($p < 0.05$)

Supplemental Table 7. Antioxidant capacity of *in vitro* digested-fermented fish depending on the fish type.

Sample	DPPH (mmol Trolox equivalents/Kg food)		
	**Digested fraction**	**Fermented fraction**	**Total antioxidant capacity**
Cod fish	$6.00^{a} \pm 6.26$	$232^{a} \pm 43.3$	$238^{a} \pm 44.1$
Salmon	$16.7^{b} \pm 5.79$	$202^{a} \pm 23.5$	$219^{a} \pm 20.4$
Mean	*11.4 ± 6.03*	*217 ± 33.4*	*229 ± 32.3*
	**FRAP (mmol Trolox equivalents/Kg food)**		
	**Digested fraction**	**Fermented fraction**	**Total antioxidant capacity**
Cod fish	$2.20^{a} \pm 0.38$	$310^{a} \pm 96.9$	$312^{a} \pm 96.7$
Salmon	$3.16^{b} \pm 0.96$	$287^{a} \pm 100$	$290^{a} \pm 100$
Mean	*2.68 ± 0.67*	*299 ± 98.5*	*301 ± 98.4*

Different letters in the same column indicates statistically significant differences ($p < 0.05$)

Supplemental Table 8. Antioxidant capacity of *in vitro* digested-fermented meat depending on the cooking method.

Cooking technique	DPPH (mmol Trolox equivalents/Kg food)		
	**Digested fraction**	**Fermented fraction**	**Total antioxidant capacity**
Boiled	$13.2^{a} \pm 5.11$	$474^{a} \pm 311$	$488^{a} \pm 314$
Fried	$19.8^{a} \pm 9.21$	$457^{a} \pm 303$	$477^{a} \pm 296$
Grilled	$10.6^{a} \pm 3.35$	$450^{a} \pm 288$	$559^{a} \pm 268$
Roasted	$16.1^{a} \pm 5.52$	$470^{a} \pm 291$	$487^{a} \pm 290$
	**FRAP (mmol Trolox equivalents/Kg food)**		
	**Digested fraction**	**Fermented fraction**	**Total antioxidant capacity**
Boiled	$2.65^{a} \pm 0.68$	$288^{a} \pm 95.0$	$290^{a} \pm 95.0$
Fried	$3.62^{a} \pm 1.21$	$263^{a} \pm 14.9$	$267^{a} \pm 15.7$
Grilled	$3.26^{a} \pm 0.39$	$323^{a} \pm 108$	$325^{a} \pm 119$
Roasted	$3.39^{a} \pm 0.74$	$277^{a} \pm 57.7$	$280^{a} \pm 57.7$

Different letters in the same column indicates statistically significant differences ($p < 0.05$)

Supplemental Table 9. Antioxidant capacity of *in vitro* digested-fermented meat depending on the meat type.

Sample	DPPH (mmol Trolox equivalents/Kg food)		
	**Digested fraction**	**Fermented fraction**	**Total antioxidant capacity**
Beef	$20.5^{a} \pm 10.8$	$175^{a} \pm 16.5$	$201^{a} \pm 19.4$
Chicken	$13.2^{a} \pm 8.94$	$196^{a} \pm 22.1$	$209^{a} \pm 16.2$
Lamb	$13.2^{a} \pm 3.12$	$745^{b} \pm 31.1$	$759^{b} \pm 31.2$
Pork	$15.2^{a} \pm 2.67$	$735^{b} \pm 52.7$	$750^{b} \pm 54.5$
Mean	*15.5 ± 3.4*	*463 ± 320*	*480 ± 317*
	**FRAP (mmol Trolox equivalents/Kg)**		
	**Digested fraction**	**Fermented fraction**	**Total antioxidant capacity**
Beef	$3.31^{a} \pm 1.55$	$306^{a,b} \pm 95.0$	$309^{a,b} \pm 102$
Chicken	$3.12^{a} \pm 0.55$	$331^{b} \pm 89.8$	$335^{b} \pm 89.8$
Lamb	$2.86^{a} \pm 0.80$	$228^{a} \pm 15.3$	$231^{a} \pm 14.9$
Pork	$3.64^{a} \pm 0.38$	$285^{a,b} \pm 49.9$	$289^{a,b} \pm 50.1$
Mean	*3.23 ± 0.33*	*288 ± 43.9*	*291 ± 44.2*

Different letters in the same column indicates statistically significant differences ($p < 0.05$)

Supplemental Table 10. Antioxidant capacity of *in vitro* digested-fermented red and white meat.

Other meats	DPPH (mmol Trolox equivalents/Kg food)		
	**Digested fraction**	**Fermented fraction**	**Total antioxidant capacity**
Red meat	$16.3^{a} \pm 7.99$	$460^{a} \pm 296$	$520^{a} \pm 287$
White meat	$14.2^{a} \pm 5.88$	$466^{a} \pm 281$	$480^{a} \pm 282$
Mean	*15.3 ± 1.48*	*463 ± 4.24*	*500 ± 28.3*
	**FRAP (mmol Trolox equivalents/Kg food)**		
	**Digested fraction**	**Fermented fraction**	**Total antioxidant capacity**
Red meat	$3.06^{a} \pm 1.15$	$267^{a} \pm 77.1$	$269^{a} \pm 73.8$
White meat	$3.37^{a} \pm 0.53$	$308^{a} \pm 74.2$	$312^{a} \pm 74.1$
Mean	*3.20 ± 0.22*	*288 ± 29.0*	*291 ± 30.4*

Different letters in the same column indicates statistically significant differences ($p < 0.05$)
